# Transdiagnostic Internet-Delivered Cognitive Behavioral Therapy for Symptoms of Postpartum Anxiety and Depression: Feasibility Randomized Controlled Trial

**DOI:** 10.2196/37216

**Published:** 2022-09-06

**Authors:** Victoria Suchan, Vanessa Peynenburg, David Thiessen, Marcie Nugent, Blake Dear, Nickolai Titov, Heather Hadjistavropoulos

**Affiliations:** 1 Department of Psychology University of Regina Regina, SK Canada; 2 Department of Mathematics and Statistics University of Regina Regina, SK Canada; 3 eCentre Clinic Department of Psychology Macquarie University Sydney Australia

**Keywords:** postpartum depression, postpartum anxiety, internet-delivered cognitive behavioral therapy, transdiagnostic, therapist assistance

## Abstract

**Background:**

Postpartum depression (PPD) and postpartum anxiety (PPA) are often comorbid and are associated with significant personal and economic costs. Fewer than half of the mothers experiencing PPD or PPA symptoms receive face-to-face treatment, suggesting a need for alternative delivery formats such as internet-delivered cognitive behavioral therapy (ICBT).

**Objective:**

This pilot study aimed to examine the impact of a therapist-assisted, transdiagnostic ICBT program on symptoms of PPD and PPA, as there is only one previous study on transdiagnostic ICBT with this population, which did not include therapist assistance.

**Methods:**

Clients endorsing the symptoms of PPD or PPA (N=63) were randomized to an 8-week transdiagnostic ICBT course (*Wellbeing Course for New Moms*) or to treatment as usual (TAU). Clients completed measures of depression, anxiety, stress, postnatal bonding, and relationship satisfaction, as well as measures of treatment satisfaction and therapeutic alliance, before treatment, after treatment, and at the 1-month follow-up. Outcome measures were also completed at the 6-month follow-up for clients who completed the ICBT course.

**Results:**

Both the ICBT and TAU groups experienced statistically significant improvements over time. The ICBT group experienced larger improvements after treatment and at the 1-month follow-up on more measures than the TAU group, with medium between-group Cohen *d* effects on primary outcome measures for anxiety (Cohen *d*=0.65, 95% CI 0.13-1.17), PPD (Cohen *d*=0.52, 95% CI 0.01-1.04), and depression (Cohen *d*=0.56, 95% CI 0.05-1.08), and on secondary outcome measures of overall distress (Cohen *d*=0.69, 95% CI 0.17-1.21), anxiety (Cohen *d*=0.59, 95% CI 0.07-1.11), and stress (Cohen *d*=0.76, 95% CI 0.23-1.28). Time-by-group interactions for proportional reductions between groups over time were only significant after treatment and at the 1-month follow-up for the primary anxiety measure (*P*=.006). This study was underpowered for detecting small or medium effects. Overall, clients perceived the treatment as credible, and 95% (21/22) of the clients were satisfied with the treatment content and therapist support.

**Conclusions:**

Findings from this pilot study provide preliminary support for transdiagnostic ICBT in treating PPD and PPA symptoms to improve access to psychological treatments.

**Trial Registration:**

ClinicalTrials.gov NCT04012580; https://clinicaltrials.gov/ct2/show/NCT04012580

## Introduction

### Background

In the period following childbirth, women are at an increased risk of experiencing depression and anxiety [1], with up to 20% of women experiencing clinical levels of symptoms [2]. To date, the vast majority of postpartum mental health research has focused on postpartum depression (PPD), leaving postpartum anxiety (PPA) poorly understood and underresearched, despite the high levels of comorbidity between these concerns [3]. The detrimental impacts associated with untreated PPD and PPA, such as impairments in mother-infant bonding [4] and infant development [5] and high economic costs [6], underscore the importance of efficacious treatment options. Cognitive behavioral therapy (CBT) has the largest evidence base for treating symptoms of PPD and PPA [7], and historically, researchers have taken a disorder-specific approach to treatment wherein they focus on a singular disorder in their trials (eg, PPD). Unfortunately, even with effective treatment options available, less than half of the new mothers experiencing symptoms seek treatment [8] because of a myriad of barriers (eg, childcare difficulties, time concerns, stigma, and transportation difficulties [9]). To address the gap between those who require and receive treatment, it is worthwhile to consider alternative treatment modalities such as internet-delivered CBT (ICBT).

### ICBT for PPA and PPD Symptoms

In ICBT, clients work through structured web-based psychoeducational and therapeutic materials similar to what would be covered in face-to-face CBT [10]. ICBT is an effective treatment for a range of mood and anxiety disorders, yielding similar effects to face-to-face CBT [11]. The delivery of ICBT can vary across several dimensions, including the type of therapist guidance (self-guided vs therapist-assisted) and specificity of content (disorder-specific vs transdiagnostic). In a meta-analysis of ICBT for nonpostpartum clients, therapist-assisted programs were superior to self-guided programs [12]. Although a growing body of literature exists demonstrating the effectiveness of ICBT in treating symptoms of anxiety [13] and depression [14] in pregnant women, as well as depression in the postpartum period [8,15,16], there is much less research on PPA. There is only one known ICBT study that specifically recruited postpartum women experiencing anxiety [17]; unfortunately, in the treatment group, only 2 treatment participants accessed all modules, and only 17% completed posttreatment questionnaires. Although this study provides helpful information, its limitations suggest that these results should be interpreted with caution.

The other notable gap in the literature is that the research on PPA and PPD symptoms has historically been disorder specific in nature (ie, recruiting women with, and providing interventions specifically for, PPA or PPD); however, the high rates of comorbidity between PPD and PPA symptoms [3] suggest that transdiagnostic ICBT may be particularly beneficial in addressing comorbid concerns efficiently. To date, only 1 study has used a transdiagnostic ICBT framework to address the symptoms of PPD or PPA [18]. The *MUMentum Postnatal* program was adapted from an existing ICBT program for adults with anxiety and depression. Adaptations included shortening the number of lessons and course duration, modifying psychoeducation throughout to be specifically relevant to postpartum concerns, and providing additional postpartum-relevant resources to accompany each lesson. The program comprised 3 core lessons tailored to new mothers. The program was intended to be completed over a span of 4 weeks and was entirely self-guided, with therapists only contacting new mothers if there were significant elevations in symptoms or suicidality. New mothers (N=87) were randomly assigned to receive either the *MUMentum* *Postnatal* program or treatment as usual (TAU). After treatment and at follow-up, new mothers assigned to the treatment group experienced larger improvements in the measures of anxiety, depression, and distress than the new mothers who received TAU. Furthermore, there were moderate but significant differences in measures of bonding and quality of life in favor of the *MUMentum Postnatal* program over TAU. Adherence rates were high, with 75% of the new mothers completing all 3 lessons, and new mothers were satisfied with the treatment overall. This trial provides preliminary evidence for the efficacy and acceptability of self-guided transdiagnostic ICBT for PPD and PPA. Additional research examining the inclusion of therapist assistance in transdiagnostic ICBT for PPD and PPA is warranted, given the finding that programs with therapist assistance often facilitate greater symptom reduction than self-guided programs [12].

### Objectives

The aim of this pilot study, which was planned before the abovementioned study was published, was to examine the efficacy of a therapist-assisted transdiagnostic program (ie, the *Wellbeing Course for New Moms*) for new mothers experiencing symptoms of PPD or PPA when delivered by a clinic specializing in the provision of ICBT and providing services on a continuous basis funded by the government. Examining the program in a routine care setting allows for greater generalizability of findings outside the initial research setting in which a program is developed [19,20]. Specifically, this study compared the effects of the *Wellbeing Course for New Moms* with TAU in reducing symptoms of anxiety and depression and improving new mothers’ rated stress, bonding with their infants, and relationship satisfaction. We took an approach similar to that of Loughnan et al [18], in that a pre-existing ICBT program was modified for new mothers, in this case, by adding an additional supplementary resource. The *MUMentum Postnatal* and *Wellbeing Course for New Moms* programs differ in terms of length (4 weeks vs 8 weeks), number of lessons (3 lessons vs 5 lessons), therapist support (unguided vs therapist guided), and specific outcome measures (eg, for distress). Another objective of this study was to examine new mothers’ satisfaction with the course. The results of this study can also serve as a replication of the findings of the *MUMentum Postnatal* program [18], which is important for establishing the generalizability of ICBT for new mothers in different settings [21]. It was hypothesized that new mothers who received the *Wellbeing Course for New Moms* would show greater improvements in anxiety, depression, stress, bonding with their infants, and relationship satisfaction and that, overall, new mothers would rate the intervention as acceptable. Given the dearth of research examining transdiagnostic ICBT tailored to new mothers, this pilot study offers important insights into how web-based interventions can help address the mental health needs of new mothers.

## Methods

### Study Context and Design

This pilot study was conducted through the Online Therapy Unit at the University of Regina. The Online Therapy Unit is a publicly funded specialized clinic that offers free ICBT to residents of Saskatchewan experiencing symptoms of anxiety or depression. The clients were randomized to receive the *Wellbeing Course for New Moms* or to TAU.

### Ethics Approval

Ethics approval was obtained from the University of Regina Ethics Board (approval number: 2019-077), and the trial was registered at ClinicalTrials.gov (NCT04012580). Participants provided consent at various points during the study, including completing a consent form before filling out the web-based screening, providing verbal consent during the telephone screening, and before initiating the *Wellbeing Course for New Moms.*

### Participants and Recruitment

Clients were recruited through web-based advertisements, presentations to health care professionals, and printed promotional materials over a span of 6 months (September 2019 to March 2020). Clients began by visiting the Online Therapy Unit website, where they completed a web-based consent form, initial eligibility questions, and a web-based screening questionnaire. To be eligible for this study, potential clients had to (1) be aged ≥18 years, (2) be female, (3) have given birth and have a child aged <1 year, (4) have a score ≥10 on the Edinburgh Postnatal Depression Scale (EPDS; [22]) or score ≥9 on the 7-item Generalized Anxiety Disorder (GAD-7) questionnaire [23], (5) be a resident of Saskatchewan, (6) be comfortable using technology, (7) have access to a secure computer and the internet, and (8) be willing to provide a medical contact as an emergency contact. Potential clients were excluded if they did not meet the abovementioned inclusion criteria or if they (1) were hospitalized in the prior year for mental health concerns or suicidality; (2) had unmanaged alcohol or drug use, mania, or psychosis; or (3) started a new psychotropic medication within the past month.

Clients who met the initial eligibility criteria completed a telephone intake interview with a doctoral student in clinical psychology (VS) or a master’s level social worker (KA). Immediately after being accepted into the trial, clients were randomly assigned to ICBT or TAU. Before recruitment for this study, the primary investigator (VS) generated a random allocation sequence using a randomizer website and specified a 1:1 ratio in blocks of 50. The sequence was produced in the form of a Microsoft Excel file, which was then uploaded directly to the website hosting the web-based screening (REDCap [Research Electronic Data Capture]; Vanderbilt University). After this point, the researchers were unable to view the allocation sequence. Randomization was performed after participants were deemed eligible for this study to limit the possibility of bias in conducting the telephone screen in the event that the randomization group was known. At the end of the telephone screen, the screener informed participants of their allocation.

### Intervention

The *Wellbeing Course for New Moms* is a transdiagnostic course based on the principles of CBT. It was adapted from the *Wellbeing Course* [24] to reflect the common concerns faced by new mothers. Clients accessed the course through the Online Therapy Unit web platform. The content of any messages and questionnaire responses were encrypted using Advanced Encryption Standard encryption with 256-bit key length. Clients completed 5 web-based lessons spanning over the course of 8 weeks, with weekly support from a therapist. The lessons resemble a Microsoft PowerPoint presentation, which participants can read through independently and move through at their own pace. Lesson 1 (1 week) provides psychoeducation about anxiety and depression in general and in postpartum populations; description of symptoms; and explanation of the relationship between unhelpful thoughts, physical symptoms, and unhelpful behaviors. Lesson 2 (2 weeks) provides information on unhelpful thoughts in relation to the CBT model and strategies for monitoring and challenging thoughts. Lesson 3 (1 week) comprises psychoeducation on physical symptoms in relation to the CBT model and strategies for managing both under- and overarousal (eg, controlled breathing). Lesson 4 (2 weeks) focuses on information related to unhelpful behaviors in relation to the CBT model and guidelines about behavioral activation and graded exposure. The fifth and final lesson (2 weeks) includes information about relapse prevention, normalization, and creation of relapse prevention plans. Each lesson includes case stories and do-it-yourself guides that were modified to be relevant to new mothers to promote the practice of strategies from the course, as well as additional resources that could be accessed at any point throughout the course (ie, assertiveness, managing beliefs, communication, mental skills, managing panic attacks, managing posttraumatic stress disorder, sleep hygiene, problem-solving and worry time, and balancing new motherhood). A new resource (ie, balancing new motherhood) was created for the purpose of this study to specifically provide information about PPD and PPA, as well as common struggles that new mothers face (eg, limited sleep, new roles, isolation, and low self-esteem). The resource was created by one of the authors (VS) and then revised before use based on feedback from the coauthor (HH), 2 psychologists with young children, and 8 mothers recruited from the community.

### Therapist Support

All clients assigned to ICBT received weekly therapist support from a master-level, registered social worker trained in the provision of ICBT. The therapist contacted clients on the same day each week throughout the 8-week course using secure emails on the Online Therapy Unit’s platform. In this way, communication between the client and therapist was asynchronous, meaning that the sender’s message may not be read until several days later by the receiver. Each therapist message was personalized but included several important elements: warmth and concern, feedback on symptom measures, highlighting key skills, answering client questions about the lessons, acknowledging any stated areas of difficulty, providing encouragement reinforcing progress, managing risk, and reminding clients about course instructions (see the study by Hadjistavropoulos et al [25] for more details). Phone calls were made to clients in specific cases (ie, heightened risk of suicide, significant increase in symptoms of anxiety or depression, or to clarify client concerns). The clients also received automated messages as reminders of new lessons or questionnaires to complete. Clients received an average of 9.89 (SD 1.03) messages and 1.93 (SD 1.96) phone calls from their therapists and sent an average of 4.32 (SD 5.23) messages.

### Measures

Outcome measures were administered before treatment, after treatment, and at the 1-month follow-up. Clients who were assigned to ICBT also completed the outcome measures at the 6-month follow-up. The measures took approximately 15 minutes to complete.

#### Primary Measures

##### EPDS Questionnaire

The EPDS [22] is a psychometrically sound 10-item self-report questionnaire that assesses symptoms of depression and anxiety in new mothers. Total scores on the EPDS range from 0 to 30, with higher scores representing more severe symptoms. Consistent with previous research [17], a cutoff of ≥10 was considered suggestive of clinical levels of depressive symptoms. The α range for the EPDS in the current trial was .86 to .97.

##### GAD-7 Measure

The GAD-7 [23] is a psychometrically sound 7-item measure that assesses symptoms of generalized anxiety and has been validated in perinatal samples [26]. Total scores range from 0 to 21, with higher scores indicating more severe symptoms of generalized anxiety. A cutoff score of 9 was used, consistent with previous trials of ICBT for PPD and PPA [18]. The α range for the GAD-7 in this trial was .75 to .92.

##### 9-Item Patient Health Questionnaire

The 9-item Patient Health Questionnaire (PHQ-9; [27]) is a psychometrically sound 9-item measure that assesses depressive symptoms and has been validated in postpartum populations [28]. Total scores range from 0 to 27, with higher scores indicating more severe depressive symptoms. The α range for the PHQ-9 in this trial was .67 to .90.

#### Secondary Measures

##### Depression and Anxiety Stress Scale

The 21-item Depression and Anxiety Stress Scale (DASS-21; [29]) is a psychometrically sound measure that comprises 21 items assessing 3 subscales: depression, stress, and anxiety. The range for total scores on the DASS-21 is 0 to 63, and the range for each subscale is 0 to 21, with higher scores indicating more severe symptoms. The α range for the DASS-21 in this trial was .89 to .94.

##### Postnatal Bonding Questionnaire

The Postnatal Bonding Questionnaire (PBQ; [30]) is a psychometrically sound 25-item self-report questionnaire that assesses mothers’ difficulties with bonding, rejection and anger, anxiety about care, and the risk of abuse regarding their infant. Total scores range from 0 to 125, with higher scores indicating more difficulties with postnatal bonding. The α range for the PBQ in this trial was .72 to .91.

##### 7-Item Dyadic Adjustment Scale

The 7-item Dyadic Adjustment Scale (DAS-7; [31]) is a psychometrically sound 7-item measure that assesses perceived relationship quality. Total scores on the DAS-7 range from 0 to 36, with higher scores indicating greater perceived relationship quality. The α range for DAS-7 in this trial was .73 to .91.

#### Treatment Satisfaction, Credibility, and Working Alliance

##### Treatment Satisfaction Questionnaire

The Treatment Satisfaction Questionnaire (TSQ; [32]) was created by the eCentre Clinic and has been used in several ICBT studies [32]. Clients completed the TSQ after treatment regarding their satisfaction with the *Wellbeing Course for New Moms*. The TSQ comprises 6 questions to assess satisfaction with the treatment (*very dissatisfied* to *very satisfied*), satisfaction with the quality of the lessons and do-it-yourself guides (*very dissatisfied* to *very satisfied*), confidence in recommending the treatment to a friend (*yes* or *no*), whether the treatment was worth their time (*yes* or *no*), how participating in treatment affected their confidence to manage symptoms (*greatly reduced* to *greatly increased*), and how participating in treatment affected their motivation to seek future treatment if needed (*greatly reduced* to *greatly increased*).

##### Adverse Effects Questionnaire

After the treatment, clients were asked to indicate whether they experienced any negative effects, new psychological symptoms, or unwanted events (eg, family member death or loss of a job) during the course. If clients answered *yes* to these questions, they were asked to explain further. Clients were also asked to indicate whether that event negatively affected their participation, engagement, or benefit from the program.

##### Credibility and Expectancy Questionnaire

The Credibility and Expectancy Questionnaire (CEQ; [33]) comprises 6 items to assess beliefs about treatment credibility and expectancy. It was administered before treatment to both groups and after treatment to the ICBT treatment group. Total scores for each of the 2 factors (ie, credibility and expectancy) ranged from 3 to 27. The α range was .60 to .88 for the credibility scale and .70 to .84 for the expectancy scale of the CEQ.

##### Working Alliance Inventory-Short Revised

The Working Alliance Inventory-Short Revised [34] was administered to the ICBT treatment group after treatment. It comprises 12 items to assess the therapeutic relationship and 3 subscales that examine the bond between therapist and client, agreement on goals, and agreement on tasks in therapy. Items on each of the 3 subscales can be summed to create a total subscale score ranging from 4 to 20. The 3 subscales can be summed to create a total working alliance score ranging from 20 to 60. The α range for the Working Alliance Inventory-Short Revised in this trial was .72 to .90.

#### Health Service Use: Service Utilization Questionnaire

All clients were asked whether they used a number of different services during the previous 8-week period at the posttreatment and 1-month follow-up (ie, medical physician, psychologist or other mental health care worker, support group, psychotropic medications, naturopathic medicine, naturopathic or homeopathic procedures, or other treatments for PPD or PPA) time points. Clients in the ICBT treatment group also received these questions at the 6-month follow-up.

### Statistical Analysis

Descriptive statistics were calculated for demographic and clinical characteristics to summarize the sample and check for pretreatment differences between the conditions. Changes in outcome measures over time were modeled using generalized estimating equations (GEEs; [35]). For all GEE models, we used an exchangeable working correlation and robust “sandwich” estimates of SEs. Gamma distributions were used to accommodate the observed skewed responses. We used a log-link function, which leads to regression coefficients and hypothesis tests assuming that changes are proportional to pretreatment measures. Modeling changes as proportional reductions from pretreatment has been recommended to provide a better model fit and reduce measurement error.

To compare improvements from before treatment to after treatment and at the 1-month follow-up, we calculated proportional improvements from the pretreatment time point and within-group and between-group Cohen *d* effect sizes. Proportional improvements and within-group Cohen *d* effect sizes are also reported for the ICBT treatment group at the 6-month follow-up. Hypothesis tests of differences in improvements between groups were performed using Wald tests on the estimated time×group interaction coefficients from the GEE models.

**Figure 1:**
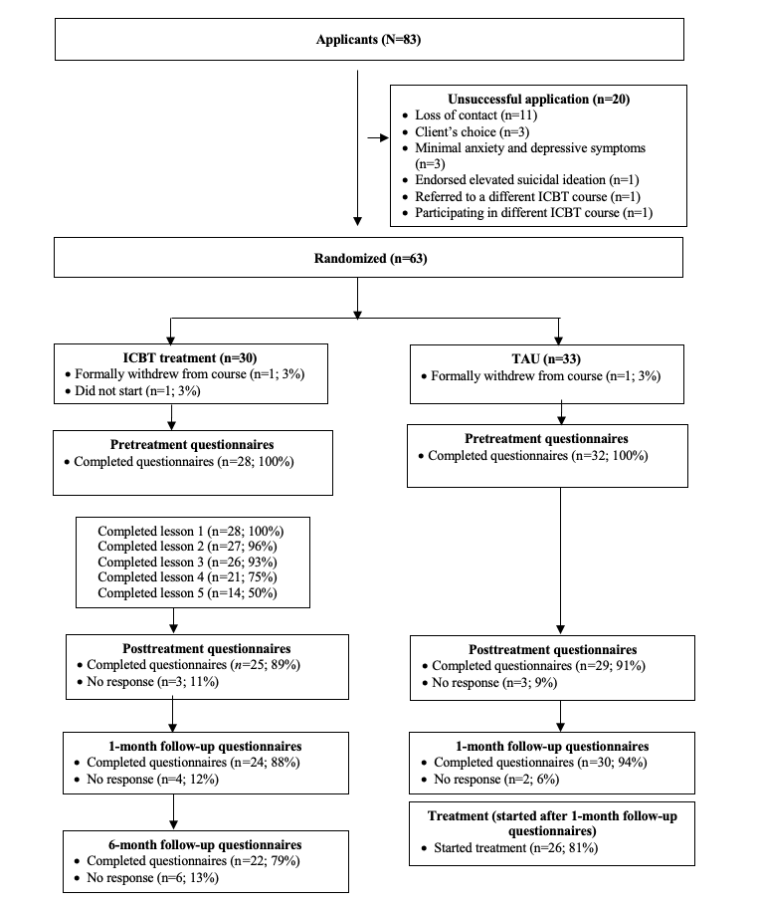
Participant flow. ICBT: internet-delivered cognitive behavioral therapy; TAU: treatment as usual.

Questionnaire completion rates were high in both groups at all observation times (Figure 1). However, ignoring missing cases can lead to an overestimation of treatment effects [36]. Therefore, we used multiple imputation to replace missing outcome measures, controlling for pretreatment symptom severity and course completion rates. In the TAU, course completion was not available; rather, we controlled for pretreatment symptom severity and whether the client accessed other mental health supports (ie, general practitioner, mental health worker, or other support groups), treating the mental health support access as a proxy measure for course engagement.

## Results

### Demographic and Clinical Characteristics

Over the 6-month recruitment period, 83 individuals applied to the *Wellbeing Course for New Moms*. Of these 83 individuals, 20 (24%) applications were considered unsuccessful for the following reasons: loss of contact (n=11, 55%), client’s choice (n=3, 15%), minimal symptoms of anxiety and depression (n=3, 15%), elevated suicidal ideation (n=1, 5%), referred to web-based materials or resources (n=1, 5%), or participating in another ICBT course (ie, *Wellbeing Course*; n=1, 5%). The remaining 63 clients were randomized to either transdiagnostic ICBT (n=30, 48%) or TAU (n=33, 52%). In the ICBT group, 3% (1/30) of clients did not start the course, and 3% (1/30) of clients formally withdrew. In the TAU group, 3% (1/33) of clients formally withdrew before completing the pretreatment questionnaires. Completion of posttreatment questionnaires (ICBT: 25/28, 89%; TAU: 29/32, 91%), 1-month follow-up questionnaires (ICBT: 24/28, 86%; TAU: 30/32, 94%), and 6-month follow-up questionnaires (ICBT: 22/28, 79%; TAU: not administered) was high. Figure 1 provides a more detailed description of the client flow.

The average age of the clients was 30.83 (SD 4.29) years. Most clients were White (51/60, 85%), married or in a common law relationship (56/60, 93%), and on maternity or parental leave (45/60, 75%). Furthermore, most clients indicated some level of postsecondary education (50/60, 83%). The average age of the clients’ infants was 4.63 (SD 3.61) months, and 60% (36/60) of the clients reported having other children. The most common type of reported birth was vaginal (40/60, 67%), followed by unplanned cesarean (12/60, 20%) or planned cesarean (8/60, 13%) sections. Additional demographic information is presented in Table 1. No statistically significant differences were found in any demographic characteristics between the ICBT treatment and TAU groups (*P*=.13-.99).

At intake, clients’ average scores on the EPDS fell within the range of probable depression (mean 14.98, SD 4.54) and within the moderate range for both the GAD-7 (mean 12.47, SD 5.27) and PHQ-9 (mean 11.33, SD 5.17). The mean scores on the PBQ and DAS-7 were 18.03 (SD 11.67) and 23.88 (SD 4.88), respectively. The only significant group difference that emerged was on the DAS-7 (t_56_=2.98; *P*=.01), whereby clients in the TAU group reported greater relationship satisfaction.

**Table 1:** Descriptive statistics for demographic variables (N=60).

Variables			All participants		ICBT^a^ (n=28)		TAU^b^ (n=32)
Age (years), mean (SD)			30.83 (4.29)		31.18 (4.31)		30.53 (4.32)
Infant’s age (months), mean (SD)			4.63 (3.12)		4.96 (2.93)		4.34 (3.13)
**Type of birth, n (%)**							
	Vaginal	40 (67)		20 (71)		20 (63)	
	Planned cesarean section	8 (13)		4 (14)		4 (13)	
	Unplanned cesarean section	12 (20)		4 (14)		8 (25)	
**Obstetric complications, n (%)**							
	No	39 (65)		17 (61)		22 (69)	
	Yes	21 (35)		11 (39)		10 (31)	
**Breastfeeding status, n (%)**							
	Breastfeeding exclusively	32 (53)		14 (50)		18 (56)	
	Breastfed initially, now using formula	14 (23)		9 (32)		5 (16)	
	Breastfeeding and formula	8 (13)		3 (11)		5 (16)	
	Formula exclusively since birth	6 (10)		2 (7)		4 (13)	
**Number of children, n (%)**							
	1	24 (40)		11 (39)		13 (41)	
	2	21 (35)		6 (21)		15 (47)	
	3	8 (13)		7 (25)		1 (3)	
	≥4	7 (12)		4 (14)		3 (9)	
**Current episode onset, n (%)**							
	Before becoming pregnant	16 (27)		9 (32)		7 (22)	
	During pregnancy	15 (25)		8 (29)		7 (22)	
	Within 1 month after birth	17 (28)		5 (18)		12 (38)	
	2-4 months after birth	5 (8)		3 (11)		2 (6)	
	5-7 months after birth	7 (12)		3 (11)		4 (13)	
**Ethnicity, n (%)**							
	White	51 (85)		21 (75)		30 (94)	
	Aboriginal (First Nations, Métis)	5 (8)		5 (18)		0	
	Other	4 (7)		2 (7)		2 (6)	
**Employment status, n (%)**							
	Working	3 (5)		1 (4)		2 (6)	
	Student	3 (5)		2 (7)		1 (3)	
	On maternity leave	45 (75)		21 (75)		24 (75)	
	Not working or on disability	9 (15)		4 (14)		5 (16)	
**Family’s annual income (US $), n (%)**							
	10,000-24,999	1 (2)		0		1 (3)	
	25,000-49,000	7 (12)		4 (14)		3 (9)	
	50,000-74,000	7 (12)		2 (7)		5 (16)	
	75,000-99,000	11 (18)		6 (21)		5 (16)	
	100,000-149,000	20 (33)		9 (32)		11 (34)	
	≥150,000	10 (17)		6 (21)		4 (13)	
	Prefer not to disclose or do not know	4 (7)		1 (4)		3 (9)	
**Education, n (%)**							
	High school diploma	11 (18)		5 (18)		6 (19)	
	College or some university	21 (35)		7 (25)		14 (44)	
	Undergraduate degree	20 (33)		8 (29)		12 (38)	
	Professional degree (eg, MD)	4 (7)		4 (14)		0	
	Graduate degree (eg, MA or PhD)	4 (7)		4 (14)		0	
**Relationship status, n (%)**							
	Married or common law	56 (93)		26 (93)		30 (94)	
	In a relationship	2 (3)		1 (4)		1 (3)	
	Separated or single	2 (3)		1 (4)		1 (3)	
**Location, n (%)**							
	Large city (>200,000)	24 (40)		11 (39)		13 (41)	
	Small city (10,000-200,000)	16 (27)		5 (18)		11 (34)	
	Town or village or farm	20 (33)		13 (43)		8 (25)	
**On psychological medication, n (%)**							
	Yes	24 (40)		13 (46)		11 (34)	
	No	36 (60)		15 (54)		21 (66)	
**Service use after treatment^c^ (n=51), n (%)**							
	Medical physician	14 (27)		4 (18)		10 (35)	
	Psychologist or mental health worker	14 (27)		5 (23)		9 (31)	
	Support group	9 (18)		5 (23)		4 (14)	
	Initiation of psychological medication	10 (20)		3 (14)		7 (24)	
	Naturopathic or homeopathic procedure	4 (8)		3 (14)		1 (3)	
**Service use at the 1-month follow-up^c^ (n=54), n (%)**							
	Medical physician	9 (17)		3 (13)		6 (20)	
	Psychologist or mental health worker	17 (32)		8 (33)		9 (30)	
	Support group	4 (7)		2 (7)		2 (7)	
	Initiation of psychological medication	9 (17)		4 (17)		5 (17)	
	Naturopathic or homeopathic procedure	8 (15)		4 (17)		4 (13)	
**Service use at the 6-month follow-up^c^ (n=22), n (%)**							
	Medical physician	1 (5)		1 (5)		—^d^	
	Psychologist or mental health worker	5 (23)		5 (23)		—	
	Support group	0 (0)		0 (0)		—	
	Initiation of psychological medication	0 (0)		0 (0)		—	
	Naturopathic or homeopathic procedure	1 (5)		1 (5)		—	

^a^ICBT: internet-delivered cognitive behavioral therapy.

^b^TAU: treatment as usual.

^c^Service use includes any visits for mental health over the previous 8 weeks.

^d^Not available.

### Primary Outcomes

Table 2 includes the means, SDs, and proportional reductions for the EPDS, GAD-7, and PHQ-9 for the groups from before treatment to after treatment and at the 1- and 6-month follow-ups. GEE analyses revealed that the ICBT treatment group experienced large improvements at all time points on the EPDS (proportional reductions 34%-46%; Cohen *d*=0.98-1.47), GAD-7 (proportional reductions 51%-63%; Cohen *d*=1.41-2.03), and PHQ-9 (proportional reductions 41%-56%; Cohen *d*=0.89-1.37). The TAU group experienced medium improvements after treatment and at the 1-month follow-up on the EPDS (proportional reductions 20%-25%; Cohen *d*=0.62-0.72), GAD-7 (proportional reductions 18%-30%; Cohen *d*=0.40-0.64), and PHQ-9 (proportional reductions 27%-31%; Cohen *d*=0.66-0.68). As indicated in Table 3, there were medium between-group effect sizes both after treatment and at the 1-month follow-up on all primary measures (Cohen *d*=0.52-0.65), with the ICBT treatment group having better outcomes. Hypothesis tests on time×group interactions showed that the differences in proportional improvements were only statistically significant on the GAD-7 (*P*=.006), with the ICBT treatment group having larger improvements. Tests were not significant on the EPDS (*P*=.20) or PHQ-9 (*P*=.16). Clients in the ICBT treatment group experienced further improvements on the EPDS (proportional reductions 32%-56%; Cohen *d*=0.88-2.06), GAD-7 (proportional reductions 53%-71%; Cohen *d*=1.39-2.68), and PHQ-9 (proportional reductions 41%-67%; Cohen *d*=0.79-1.95) at the 6-month follow-up.

**Table 2:** Estimated marginal means, SDs, and percentage changes for primary and secondary outcomes by group.

Outcomes				Estimated marginal means (SD)									Percentage changes from pretreatment questionnaires															
				Pretreatment		Posttreatment		1-month follow-up		6-month follow-up		Posttreatment							1-month follow-up							6-month follow-up (95% CI)		
												95% CI			*P* value			95% CI				*P* value						
**Primary outcomes**																												
	**EPDS^a^**															.13							.06					
		ICBT^b^	14.47 (4.27)		9.54 (5.59)		8.75 (4.38)		7.86 (4.58)		34 (17 to 48)						40 (27 to 50)							46 (32 to 57)				
		TAU^c^	15.44 (4.79)		12.35 (5.04)		11.54 (5.82)		—^d^		20 (8 to 31)						25 (11 to 37)							—				
	**GAD-7^e^**															.002							.006					
		ICBT	13.36 (5.00)		6.51 (4.56)		5.79 (3.90)		4.97 (2.87)		51 (36 to 63)						57 (43 to 67)							63 (53 to 71)				
		TAU	11.69 (5.46)		9.58 (4.80)		8.24 (5.26)		—		18 (2 to 31)						30 (12 to 44)							—				
	**PHQ-9^f^**															.15							.06					
		ICBT	10.68 (5.11)		6.29 (4.64)		5.53 (3.18)		4.74 (3.23)		41 (22 to 56)						48 (35 to 59)							56 (41 to 67)				
		TAU	11.91 (5.24)		8.73 (3.94)		8.23 (5.75)		—		27 (14 to 38)						31 (12 to 46)							—				
**Secondary outcomes**																												
	**DASS-21^g^ total**															.04							.02					
		ICBT	24.14 (10.44)		13.03 (9.80)		12.17 (7.64)		9.91 (7.94)		46 (26 to 61)						50 (36 to 60)							59 (44 to 70)				
		TAU	26.25 (12.85)		20.24 (10.73)		18.57 (10.09)		—		23 (7 to 36)						29 (15 to 41)							—				
	**DASS-21 Depression**															.32							.11					
		ICBT	6.83 (5.11)		4.08 (3.59)		3.96 (2.95)		2.87 (3.74)		40 (14 to 59)						42 (23 to 56)							58 (30 to 75)				
		TAU	7.57 (5.22)		5.62 (4.48)		5.98 (5.24)		—		26 (2 to 44)						21 (−8 to 42)							—				
	**DASS-21 Anxiety**															.07							.13					
		ICBT	6.08 (3.41)		2.61 (3.49)		2.45 (2.87)		1.93 (2.22)		57 (24 to 76)						60 (35 to 75)							68 (50 to 80)				
		TAU	6.57 (5.17)		4.94 (4.21)		4.00 (3.07)		—		25 (−1 to 44)						39 (21 to 53)							—				
	**DASS-21 Stress**															.03							.02					
		ICBT	11.25 (4.02)		6.61 (3.92)		5.96 (3.26)		5.17 (3.22)		41 (25 to 54)						47 (34 to 57)							54 (42 to 64)				
		TAU	12.13 (4.79)		9.68 (4.07)		8.75 (4.39)		—		20 (7 to 31)						28 (14 to 39)							—				
	**PBQ^h^**															.63							.70					
		ICBT	17.25 (10.38)		12.05 (6.97)		10.58 (4.88)		8.70 (7.43)		30 (11 to 45)						39 (26 to 49)							50 (29 to 64)				
		TAU	18.72 (12.82)		14.27 (10.03)		12.32 (9.21)		—		24 (2 to 41)						34 (15 to 49)							—				
	**DAS-7^i^**															.04							.63					
		ICBT	21.96 (3.72)		23.37 (3.85)		22.81 (4.29)		24.00 (4.78)		10 (2 to 21)						6 (−7 to 18)							15 (0 to 27)				
		TAU	25.55 (5.21)		25.09 (5.41)		25.76 (5.52)		—		−4 (−24 to 12)						2 (−18 to 19)							—				

^a^EPDS: Edinburgh Postnatal Depression Scale.

^b^ICBT: internet-delivered cognitive behavior therapy.

^c^TAU: treatment as usual.

^d^Not available.

^e^GAD-7: 7-item Generalized Anxiety Disorder.

^f^PHQ-9: 9-item Patient Health Questionnaire.

^g^DASS-21: 21-item Depression and Anxiety Stress Scale.

^h^PBQ: Postnatal Bonding Questionnaire.

^i^DAS-7: 7-item Dyadic Adjustment Scale.

**Table 3:** Within-group and between-group effect sizes for primary and secondary outcomes.

Outcomes				Within-group effect sizes from pretreatment questionnaires (95% CI)							Between-group effect sizes from pretreatment questionnaires (95% CI)		
				Posttreatment		1-month follow-up		6-month follow-up		Posttreatment			1-month follow-up
**Primary outcomes**													
	**EPDS^a^**												
		ICBT^b^	0.98 (0.42 to 1.53)		1.30 (0.73 to 1.88)		1.47 (0.88 to 2.06)		0.52 (0.01 to 1.04)			0.53 (0.01 to 1.05)	
		TAU^c^	0.62 (0.12 to 1.12)		0.72 (0.22 to 1.23)		N/A^d^		0.52 (0.01 to 1.04)			0.53 (0.01 to 1.05)	
	**GAD-7^e^**												
		ICBT	1.41 (0.83 to 2.00)		1.67 (1.06 to 2.27)		2.03 (1.39 to 2.68)		0.65 (0.13 to 1.17)			0.52 (0.00 to 1.03)	
		TAU	0.40 (–0.09 to 0.90)		0.64 (0.13 to 1.14)		N/A		0.65 (0.13 to 1.17)			0.52 (0.00 to 1.03)	
	**PHQ-9^f^**												
		ICBT	0.89 (0.34 to 1.44)		1.19 (0.62 to 1.76)		1.37 (0.79 to 1.95)		0.56 (0.05 to 1.08)			0.56 (0.05 to 1.08)	
		TAU	0.68 (0.17 to 1.18)		0.66 (0.16 to 1.16)		N/A		0.56 (0.05 to 1.08)			0.56 (0.05 to 1.08)	
**Secondary outcomes**													
	**DASS-21^g^ total**												
		ICBT	1.08 (0.52 to 1.64)		1.29 (0.72 to 1.87)		1.51 (0.92 to 2.11)		0.69 (0.17 to 1.21)			0.70 (0.18 to 1.22)	
		TAU	0.50 (0.00 to 1.00)		0.66 (0.15 to 1.16)		N/A		0.69 (0.17 to 1.21)			0.70 (0.18 to 1.22)	
	**DASS-21 Depression**												
		ICBT	0.62 (0.08 to 1.15)		0.68 (0.14 to 1.22)		0.87 (0.32 to 1.42)		0.37 (−0.14 to 0.88)			0.46 (−0.05 to 0.98)	
		TAU	0.40 (−0.10 to 0.89)		0.30 (−0.19 to 0.79)		N/A		0.37 (−0.14 to 0.88)			0.46 (−0.05 to 0.98)	
	**DASS-21 Anxiety**												
		ICBT	0.99 (0.44 to 1.55)		1.14 (0.57 to 1.70)		1.42 (0.84 to 2.01)		0.59 (0.07 to 1.11)			0.51 (0.00 to 1.03)	
		TAU	0.34 (−0.15 to 0.84)		0.60 (0.10 to 1.10)		N/A		0.59 (0.07 to 1.11)			0.51 (0.00 to 1.03)	
	**DASS-21 Stress**												
		ICBT	1.15 (0.59 to 1.72)		1.42 (0.84 to 2.01)		1.65 (1.04 to 2.25)		0.76 (0.23 to 1.28)			0.70 (0.18 to 1.23)	
		TAU	0.54 (0.04 to 1.04)		0.73 (0.22 to 1.23)		N/A		0.76 (0.23 to 1.28)			0.70 (0.18 to 1.23)	
	**PBQ^h^**												
		ICBT	0.58 (0.05 to 1.11)		0.81 (0.27 to 1.36)		0.93 (0.38 to 1.49)		0.25 (−0.26 to 0.76)			0.23 (−0.28 to 0.74)	
		TAU	0.38 (−0.11 to 0.88)		0.57 (0.07 to 1.07)		N/A		0.25 (−0.26 to 0.76)			0.23 (−0.28 to 0.74)	
	**DAS-7^i^**												
		ICBT	0.37 (−0.16 to 0.90)		0.21 (−0.32 to 0.73)		0.47 (−0.06 to 1.00)		−0.36 (−0.87 to 0.15)			−0.58 (−1.10 to −0.07)	
		TAU	−0.09 (−0.58 to 0.40)		0.04 (−0.45 to 0.53)		N/A		−0.36 (−0.87 to 0.15)			−0.58 (−1.10 to −0.07)	

^a^EPDS: Edinburgh Postnatal Depression Scale.

^b^ICBT: internet-delivered cognitive behavior therapy.

^c^TAU: treatment as usual.

^d^N/A: not applicable.

^e^GAD-7: 7-item Generalized Anxiety Disorder.

^f^PHQ-9: 9-item Patient Health Questionnaire.

^g^DASS-21: 21-item Depression and Anxiety Stress Scale.

^h^PBQ: Postnatal Bonding Questionnaire.

^i^DAS-7: 7-item Dyadic Adjustment Scale.

### Secondary Outcomes

Means, SDs, and proportional reductions for all the secondary measures are shown in Table 2. The ICBT treatment group showed large improvements on the total DASS-21 and on the DASS-21 Anxiety and Stress subscales, large to medium improvements on the DASS-21 Depression subscale and the PBQ, and small improvements on the DAS-7 after treatment and at the 1- and 6-month follow-ups (Table 2). The TAU group had medium improvements on the DASS-21 and DASS-21 Stress subscale, medium to small improvements on the DASS-21 Anxiety subscale and the PBQ, small improvements on the DASS-21 Depression subscale, and negligible changes on the DAS-7. Between-group Cohen *d* effect sizes were medium on the DASS-21, DASS-21 Anxiety subscale, and DASS-21 Stress subscale; medium to small on the DAS-7; and small on the DASS-21 Depression subscale and the PBQ (Table 3). The between-group Cohen *d* effect sizes favored the ICBT treatment group on all measures except the DAS-7, where the TAU group had better scores after treatment and at the 1-month follow-up. However, interpretation of the DAS-7 is complicated by the fact that the groups had significantly different averages before treatment.

Hypothesis tests on the time×group interactions showed that the differences in proportional improvements were statistically significant for the DASS-21 (*P*=.04), DASS-21 Stress subscale (*P*=.03), and the DAS-7 after treatment (*P*=.04) but not at the 1-month follow-up. Differences in proportional reductions were not significant after treatment or at the 1-month follow-up on the DASS-21 Anxiety subscale, DASS-21 Depression subscale, or the PBQ.

### Service Use

Service use after treatment and at the 1-month and 6-month follow-ups is summarized in Table 1. Between-group comparisons found no significant differences in health service use (*P*=.18-.99). Comparisons were not possible at the 6-month follow-up as only treatment clients answered these questions.

### Treatment Adherence, Acceptability, and Satisfaction

Table 4 includes details about treatment adherence, acceptability, and satisfaction. In the ICBT treatment group, 75% (21/28) of the participants completed at least four of the five lessons, and 50% (14/28) of the participants completed all 5 lessons. Before treatment, the mean score on the credibility factor of the CEQ was 21.22 (SD 3.38), and the mean score on the expectancy factor was 17.07 (SD 3.77), with no significant differences between the ICBT treatment and TAU groups (*P*=.59 and *P*=.44 on the credibility and expectancy factors, respectively). Clients in the ICBT treatment group demonstrated a significant increase in treatment credibility scores after treatment (mean 23.58, SD 3.02; t_21_=2.66; *P*<.05).

**Table 4:** Intervention use and treatment satisfaction (N=22).

Variables		Values
**Intervention use, mean (SD; range)**		
	Number of log-ins	22.25 (13.81; 5-73)
	Messages sent to the therapist	5.32 (5.23; 0-29)
	Messages received from the therapist	9.89 (1.03; 8-12)
	Number of phone calls from the therapist	1.93 (1.96; 0-6)
	Furthest lesson accessed	4.57 (0.78; 2-5)
**Working alliance (WAI-SR^a^; mean [SD; range])**		
	Total	47.77 (8.65; 22.00-58.00)
	Goal subscale	15.36 (3.74; 5.00-20.00)
	Task subscale	16.27 (2.00; 11.00-20.00)
	Bond subscale	16.14 (3.67; 6.00-20.00)
**Treatment satisfaction, n (%)**		
	Would recommend the course to a friend	22 (100)
	The course was worth their time	22 (100)
	Satisfied or very satisfied with the treatment	21 (95)
	Satisfied or very satisfied with course materials	20 (91)
	Increased or greatly increased confidence in managing symptoms	21 (95)
	Increased or greatly increased motivation to seek additional treatment if needed in the future	17 (77)

^a^WAI-SR: Working Alliance Inventory-Short Revised.

## Discussion

### Principal Findings

In this study, 63 new mothers were randomized to receive either the *Wellbeing Course for New Moms* (ICBT) or TAU (ie, continued access to their standard care providers). Medium between-group Cohen *d* effects were observed in favor of the ICBT condition on most measures of anxiety and depression after treatment and at the follow-ups, with the exception of the depression subscale of the DASS-21. However, when examining the time-by-group interaction comparing differences in proportional reductions from before treatment between ICBT and TAU, the differences were only significant for the GAD-7 both after treatment and at the 1-month follow-up. Owing to the small sample size, this study was underpowered to detect significant differences in recovery rates between the treatment and TAU conditions, which may help explain the difference in findings based on effect sizes compared with time-by-group interactions of the proportional reductions. Improvements on all primary (EPDS, GAD-7, and PHQ-9) and most secondary measures (DASS-21 and PBQ) were maintained at the 6-month follow-up in the ICBT group. Finally, clients were satisfied with the ICBT course, perceived it as credible, and expressed high levels of alliance with their therapists.

The symptom improvement demonstrated in this sample is consistent with the treatment responses found in the face-to-face CBT literature. Meta-analyses have found statistically significant effects of CBT in the treatment of PPD, and improvements were maintained at the 6-month follow-up [37,38]. Although no known individual CBT studies have specifically investigated PPA, studies that report on comorbid anxiety symptoms [39,40] report that CBT is also effective in improving PPA symptoms. In terms of other ICBT trials, the *MUMentum Postnatal* program [18] is the only other published trial of transdiagnostic ICBT for the symptoms of PPD and PPA. In that trial, large between-group differences were found in favor of ICBT over TAU on measures of anxiety, depression, and distress after treatment and at follow-up. In this trial, medium between-group effects were found in favor of ICBT on all primary outcome measures (EPDS, PHQ-9, and GAD-7). Despite medium between-group effects, the differences in proportional improvements were only significant for the GAD-7 both after treatment and at the 1-month follow-up. Two notable differences between the trials included the proportion of clients who were referred by a medical or mental health professional (60% in this trial vs 6% in the study by Loughnan et al [18]) and the proportion of patients taking medication before treatment (40% in this trial vs 8% in the study by Loughnan et al [18]), although differences may have emerged because of the exclusion criteria for each study. Most clients in the study by Loughnan et al [18] self-referred to the program, suggesting that they may have been more motivated to complete the treatment, potentially resulting in better outcomes. Furthermore, the *MUMentum Postnatal* program contained more psychoeducation relevant to new mothers throughout the program (as opposed to a stand-alone resource) and is a briefer, more condensed treatment than *the Wellbeing Course for New Moms*, which may be more appropriate for this population, given the time constraints of caring for a new infant.

### Strengths

Given the scarcity of published trials examining ICBT for PPA and PPD symptoms, this pilot study makes an important contribution to the literature [41]. A strength of this study is that it examined a transdiagnostic intervention as opposed to a disorder-specific intervention, which allows new mothers to address comorbid symptoms simultaneously instead of requiring separate ICBT courses. Furthermore, this trial provided helpful information about the trajectory of PPA and PPD symptoms among new mothers who were assigned to the TAU group. Specifically, new mothers in the TAU group reported significant improvements across primary measures, suggesting that symptoms of PPA and PPD may decrease in the absence of ICBT or perhaps that knowledge of upcoming treatment can facilitate symptom change.

### Limitations and Future Directions

Despite the contributions of this pilot study to the literature on ICBT for symptoms of PPA and PPD, this study had several limitations that should be considered when interpreting the results. Owing to the timing of the trial, approximately half of the clients were enrolled in the *Wellbeing Course for New Moms* during the COVID-19 pandemic; of note, research indicates that the COVID-19 pandemic has led to a substantial increase in the rates of PPD and PPA [42]. Clients commented on some of the challenges of engaging with ICBT during the pandemic and noted that opportunities to practice strategies (eg, pleasant activity scheduling or graded exposure) may have been limited by public health recommendations for physical distancing and self-isolation. It will be important to assess whether the findings from this study are replicated after the pandemic. An additional limitation of this pilot study was its small sample size, which meant that it was not reliably powered to detect small to medium between-group effects. Furthermore, the small sample size limited the types of analyses that could be conducted and prevented us from being able to determine moderators of symptom change (eg, relationship satisfaction, symptom severity and onset, use of medication, and access to other mental health supports). Future trials of the *Wellbeing Course for New Moms* should include larger sample sizes to ensure sufficient power for additional analyses. In future trials, it would be interesting to explore the extent to which individuals with PPA or PPD use additional resources. Finally, this study relied on self-report symptom measures as opposed to engaging participants in structured clinical interviews to ascertain information about their symptoms. Although this is not uncommon in the ICBT literature, it resulted in a lack of clarity regarding the participants’ diagnostic status.

### Conclusions

This pilot study is the first known study of therapist-assisted, transdiagnostic ICBT designed to target symptoms of PPD and PPA. We found that the treatment resulted in larger improvements in depression, anxiety, and overall distress relative to a TAU condition when examining between-group effect sizes. However, the time-by-group interaction was significant only for the GAD-7 both after treatment and at the 1-month follow-up. Improvements in primary outcomes in the ICBT group were maintained at the 6-month follow-up. This study provides initial evidence for transdiagnostic ICBT as a potentially accessible, effective, and acceptable treatment for symptoms of PPD and PPA.

## Appendix

Multimedia Appendix 1 CONSORT eHEALTH checklist (V 1.6.1).

## Abbreviations

CBT: cognitive behavioral therapy
CEQ: Credibility and Expectancy Questionnaire
DAS-7: 7-item Dyadic Adjustment Scale
DASS-21: 21-item Depression and Anxiety Stress Scale
EPDS: Edinburgh Postnatal Depression Scale
GAD-7: 7-item Generalized Anxiety Disorder
GEE: generalized estimating equation
ICBT: internet-delivered cognitive behavioral therapy
PBQ: Postnatal Bonding Questionnaire
PHQ-9: 9-item Patient Health Questionnaire
PPA: postpartum anxiety
PPD: postpartum depression
REDCap: Research Electronic Data Capture
TAU: treatment as usual
TSQ: Treatment Satisfaction Questionnaire
